# Current and future availability of and need for human resources for sexual, reproductive, maternal and newborn health in 41 countries in Sub-Saharan Africa

**DOI:** 10.1186/s12939-017-0569-z

**Published:** 2017-05-03

**Authors:** Maria Guerra Arias, Andrea Nove, Michaela Michel-Schuldt, Luc de Bernis

**Affiliations:** 1 Instituto de Cooperación Social Integrare, Barcelona, Spain; 2 UNFPA, New York, NY USA

**Keywords:** Midwifery, Health workforce, Evidence, Metrics, Coverage, Sub-Saharan Africa, Sexual, reproductive, maternal and newborn health

## Abstract

**Background:**

The WHO African region, covering the majority of Sub-Saharan Africa, faces the highest rates of maternal and neonatal mortality in the world. This study uses data from the State of the World’s Midwifery 2014 survey to cast a spotlight on the WHO African region, highlight the specific characteristics of its sexual, reproductive, maternal and newborn health (SRMNH) workforce and describe and compare countries’ different trajectories in terms of meeting the population need for services.

**Methods:**

Using data from 41 African countries, this study used a mathematical model to estimate potential met need for SRMNH services, defined as “the percentage of a universal SRMNH package that could potentially be obtained by women and newborns given the composition, competencies and available working time of the SRMNH workforce.” The model defined the 46 key interventions included in this universal SRMNH package and allocated them to the available health worker time and skill set in each country to estimate the potential met need.

**Results:**

Based on the current and projected potential met need in the future, the countries were grouped into three categories: (1) ‘making or maintaining progress’ (expected to meet more, or the same level, of the need in the future than currently): 14 countries including Ghana, Senegal and South Africa, (2) ‘at risk’ (currently performing relatively well but expected to deteriorate due to the health workforce not keeping pace with population growth): 6 countries including Gabon, Rwanda and Zambia, and (3) ‘low performing’ (not performing well and not expected to improve): 21 countries including Burkina Faso, Eritrea and Sierra Leone.

**Conclusion:**

The three groups face different challenges, and policy solutions to increasing met need should be tailored to the specific context of the country. National health workforce accounts should be strengthened so that workforce planning can be evidence-informed.

**Electronic supplementary material:**

The online version of this article (doi:10.1186/s12939-017-0569-z) contains supplementary material, which is available to authorized users.

## Background

The State of the World’s Midwifery (SoWMy) 2014 [1] provides an evidence base and analysis of the workforce delivering sexual, reproductive, maternal and newborn health (SRMNH) services in low- and middle-income countries with the highest rates of maternal and newborn mortality. A successor to the first SoWMy report published in 2011 [2], SoWMy 2014 explored four dimensions of “effective coverage” of SRMNH care: availability, accessibility, acceptability and quality (AAAQ). The concept of effective coverage was developed by T. Tanahashi and the World Health Organization (WHO) in the 1970’s to explore the delivery of health services [3]. The United Nations Committee on Economic, Social and Cultural Rights published General Comment No. 14 on the right to health [4], which mirrored the Tanahashi domains of availability, accessibility and acceptability, with quality as the fourth domain.

The SRMNH workforce examined in the report includes all health professionals whose primary functions involve providing services to women during pre-pregnancy, pregnancy, labour and birth, and post-partum care for mothers and newborns. This can include midwives, nurse-midwives, doctors and also other professionals with some midwifery competencies such as nurses, or auxiliary nurses and midwives [1].

To prepare this global report, the SoWMy survey collected data from 73 countries, of which 41 are in the WHO’s Africa region [5]. Covering the majority of Sub-Saharan Africa, this region faces the double burden of highest population growth rates and weakest health systems [6–8]. Although unprecedented progress has been made in reducing maternal mortality since the 1990s, rates are still much higher than the global average. The maternal mortality ratio (MMR) in Sub-Saharan Africa has dropped by 45% over the past 25 years, from 987 deaths per 100 000 live births in 1990 to 546 in 2015. However, this was still the highest MMR of any world region in 2015, and more than double the global average of 216 [9]. These global inequities in the survival of women during pregnancy and childbirth are directly at odds with the Sustainable Development Goals (SDG) agenda of ensuring healthy lives for all, and ensuring universal access to sexual and reproductive healthcare services [10]. At the same time, there are great inequities within the African region: for example, the MMR in Sierra Leone (1360) is more than ten times higher than the MMR in Botswana (129) [9].

A number of plans and strategies that are relevant to the SRMNH workforce have been developed specifically for Africa, including: the Africa Health Strategy for 2016–2030 [11], the Maputo Plan of Action for the Operationalisation of the Continental Policy Framework for Sexual and Reproductive Health and Rights [12] and the Road Map for Scaling up HRH in the African Region 2012–2025 [13]. The Africa Health Strategy calls on all African Union member states to develop a HRH management plan which addresses “policies, strategic plans, information, training, recruitment, deployment and retention, administration, working and living conditions and the health of staff”. The Road Map identifies six strategic areas that require strengthening: (1) health workforce leadership and governance capacity, (2) HRH regulatory capacity, (3) education and training of health workers, (4) optimising the utilisation, retention and performance of health workers, (5) improving health workforce information and generation of evidence for decision-making, and (6) health workforce dialogue and partnership.

Additionally, targeted programmes are focusing on improving the situation in the region, such as: (1) the Campaign for Accelerated Reduction of Maternal Mortality in Africa (CARMMA) supported by the African Union Commission, the United Nations Population Fund (UNFPA), the WHO and the United Nations Children’s Fund (UNICEF) [14], (2) the World Bank/UNFPA Sahel Women’s Empowerment and Demographics Project [15], (3) the MamaYe campaign [16], (4) the International Confederation of Midwives/Sanofi Espoir Foundation’s Strengthening Midwifery Education in French Speaking African Countries initiative [17], (5) the WHO/Muskoka work on accreditation of midwifery schools in French-speaking African countries [18], and (6) ICM’s work on implementation of the Midwifery Services Framework for developing SRMNH services [19].

The 2010 Global Strategy for Women’s and Children’s Health [20] - and the Every Woman Every Child movement that grew out of it - have been revised and updated to include adolescents’ health, and the updated Global Strategy [21] includes the period 2016 to 2030 to align with the SDG timeframe. It is expected that new financial channels dedicated to reproductive, maternal, newborn and adolescent health, like the Global Financing Facility [22], will allow health workforce challenges in high burden countries to be better addressed, when accurate and relevant workforce data are provided.

This study uses data from the SoWMy 2014 survey to cast a spotlight on the WHO African region, highlight the specific characteristics of its SRMNH workforce and describe and compare countries’ different trajectories in terms of meeting the need for SRMNH services between 2012 and 2030. It also explores regional challenges and solutions to delivering effective coverage of SRMNH services.

## Methods

### Primary data collection

Data were collected through the SoWMy 2014 survey. An online questionnaire, available in English, French and Spanish, was sent to UNFPA and WHO country offices, who were asked to engage with stakeholders such as national midwives associations and ministries of health to collect data on the national SRMNH workforce and delivery of SRMNH services. The questionnaire was sent to the 75 Countdown countries, of which 73 responded, and 41 are included in this analysis as they belong to the WHO African region (see Additional file 1 for list of countries included). The questionnaire included questions on the size and structure of the SRMNH workforce, education and career pathways, regulatory systems, professional associations and policy and planning systems. Responses received were carefully reviewed by the investigators for completeness and consistency and detailed lists of queries were returned to each country office for a second round of review and validation. A helpdesk was available throughout the process to clarify definitions and questions about the data being collected. The final data were validated by representatives of the ministry of health in each country.

### Calculating potential met need

Potential met need for essential SRMNH services in each country is defined as “the percentage of a universal SRMNH package that could potentially be obtained by women and newborns given the composition, competencies and available working time of the SRMNH workforce.” It is not an estimate of the amount of need that is actually being met - it is a measure of the potential of the workforce to meet the need, if workers were educated to international standards and the workload were to be allocated in an economically efficient manner. It is expressed as the percentage of health worker time that would be needed to deliver all essential SRMNH interventions to all in need of them that is available in the current workforce. Potential met need was calculated using a mathematical model,for which the methodology and data sources are detailed in Annexes 3 and 4 of the SoWMy report [23] and summarised below.

Before commencing the modelling, we defined the key interventions representing need for SRMNH care. For this we used the list of 46 essential interventions proposed by the Partnership for Maternal, Newborn and Child Health (PMNCH) [24]. The model itself was run separately for each country as follows:

1. Estimate how much health worker time would be required to deliver each essential intervention to everyone who needed it in the baseline year (2012), by:
  - estimating the number of women, girls and babies requiring each intervention using demographic and epidemiological data from secondary sources (see Annex 4 of the SoWMy report [23] for details).
  - estimating the contact time required to deliver each intervention to one individual, using time estimates from the OneHealth tool [25] where available, otherwise expert estimates,
  - based on the previous two quantities, estimating the total annual contact time required to deliver each intervention to all the individuals who need it,
  - translating the total annual hours required to deliver each intervention into the equivalent number of FTEs, where it was assumed that all SRMNH workers work 40 h per week, take an average of 5 days of sickness leave and 20 days of paid annual leave per year, spend 70% of their available working hours providing clinical interventions (as opposed to administrative tasks and other duties) and spend a (country-estimated) proportion of their clinical work time on SRMNH care (as opposed to other types of health care), and
  - summing the number of FTEs needed to deliver universal coverage of all the interventions.
2. Estimate the amount of health worker time that is available to meet the need. This was done by allocating the annual number of FTE workers required to deliver each intervention to the least costly cadre with the relevant competencies. This step involves:
  - converting the health worker headcounts provided by each country to FTEs as per step 1d above,
  - defining the cadre(s) that should be responsible for providing at least one essential intervention (even if they do not currently do so) and assigning them to one of seven standardized categories of health worker as per the International Standard Classification of Occupations [26], based on their years of training and functional roles, rather than on the specific cadre name in the country. This standardization allowed the model to be applied consistently across different countries. The seven categories were ordered from lowest paid to highest paid (based on analysis of salaries provided in the SoWMy survey) as follows: auxiliary nurses, auxiliary midwives, nurses, midwives, medical officers, generalist physicians, obstetrician/gynaecologists,
  - determining which of the SRMNH interventions each cadre category should be competent to perform (even if they do not currently do so) based on the International Standard Classification of Occupations [26] and Optimize MNH [27], and
  - using a logical algorithm, allocating sequentially to each cadre category (starting with the least expensive) the number of FTEs required to provide universal coverage for each intervention based on whether or not that cadre is competent to deliver that intervention. If there were insufficient FTEs in the least expensive category to meet all of the need for an intervention, the unallocated time was allocated to the next category until either all of the need had been (theoretically) met or all of the available FTE time had been allocated.

Potential met need was calculated for 2012 and projected for every year up to 2030. To carry out the projections, the evolution of the workforce between 2012 and 2030 was modelled using a ‘stock and flow’ method, which takes into account the age structure of the workforce in 2012, then adds inflows for each year (from data provided by countries about the number of new graduates entering the workforce each year) and subtracts outflows for each year due to death (based on age-specific death rates), voluntary attrition (based on country estimates) and retirement (based on each country’s statutory retirement ages). Future projections were used for demographic data such as the number of women of reproductive age, but it was assumed that the baseline epidemiological conditions would continue to apply to 2030.

The model also allowed an exploration of alternative policy scenarios, assessing the impact on potential met need between 2012 and 2030 when certain parameters of the model are altered. In this study, we compare two possible scenarios: the current trajectory, where none of the parameters of the model is altered, and the “what if?” trajectory, which models the impact of a policy scenario which combines the following four outcomes: a) reducing pregnancies by 20% by 2030; b) doubling the number of midwife, nurse and physician graduates by 2020; c) improving efficiency by 2% per year until 2030; d) halving the rate of voluntary attrition between 2012 and 2017. These ‘what if’ figures were based on a review of the literature and strategic documents with indicative targets, e.g. the FP2020 initiative’s planned increases in the contraceptive prevalence rate [28], discussions at the Third Global Forum on Human Resources for Health in 2013 [29] and WHO guidelines on transforming health professional education [30].

A summary of the model results for this study is included in the additional files (see Additional file 1).

An important consideration is that the potential met need estimates refer exclusively to the dimension of availability within the AAAQ framework. Thus, the model does not account for whether the SRMNH services provided to the population are accessible, acceptable or of high quality. The implications of this are explored in the discussion section of this article.

### Results

#### Overview. Characteristics of the workforce in the WHO African region

**Fig. 1:**
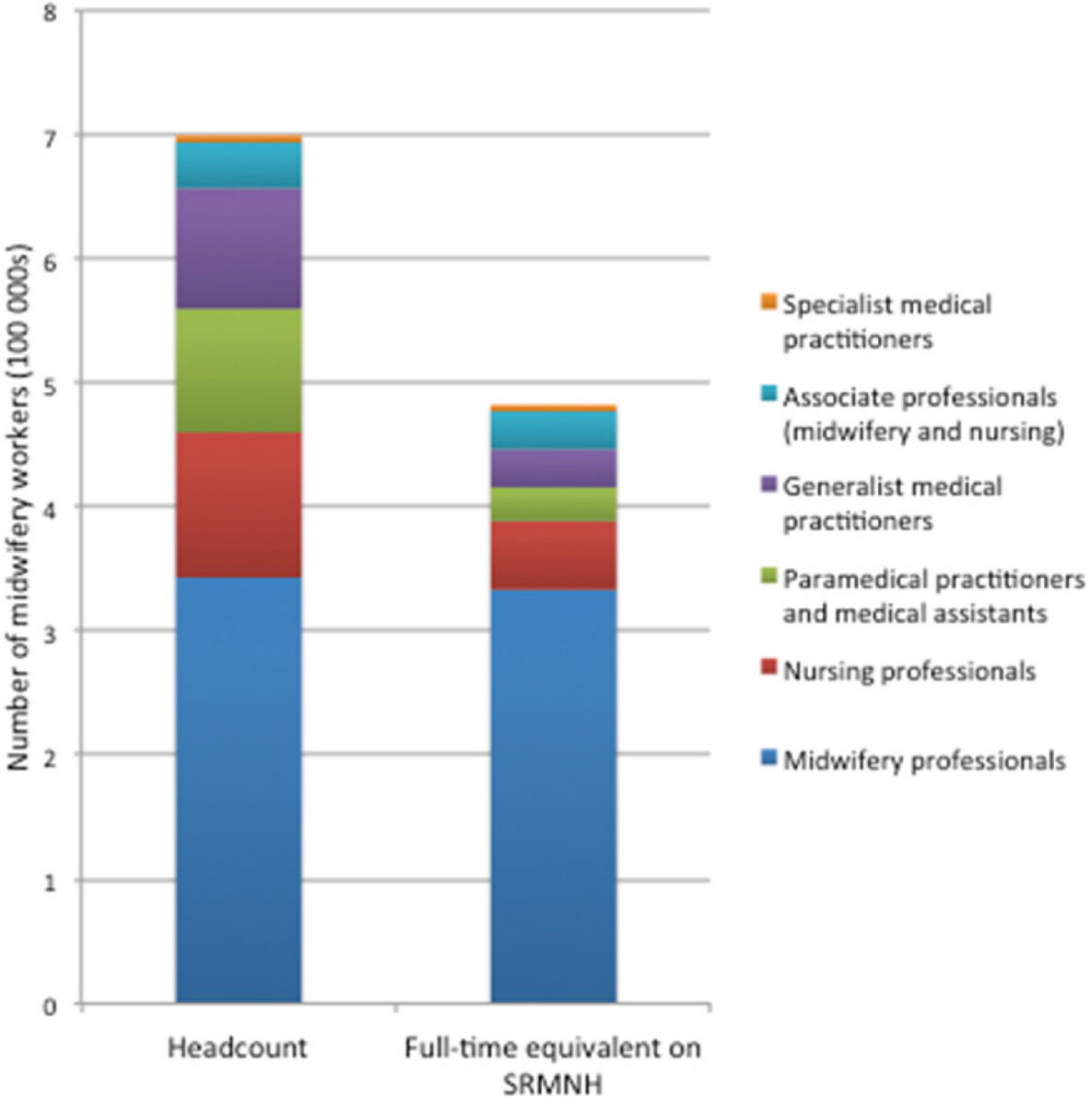
SRMNH workforce in Africa region: headcount vs. FTE on SRMNH

The 41 countries reported a total of almost 700,000 health professionals working in the field of SRMNH. The SRMNH workforce analysed includes the following ISCO categories: Generalist medical practitioners (ISCO code 2211), Specialist medical practitioners (including obstetricians/gynaecologists) (2212), Nursing professionals (2221), Midwifery professionals (2222), Paramedical practitioners such as medical officers (2240), Nursing associate professionals (3221), Midwifery associate professionals (3222), Medical assistants (3256). Availability is explored in Fig. 1. The first column shows the availability of SRMNH workers by headcount: the number of workers in each category. The second column shows the availability by full-time equivalent (FTE): calculated by multiplying the total number of health workers by the percentage of time they spend on SRMNH. These figures show the importance of taking into account the percentage time spent on SRMNH: the real “availability” of SRMNH workers is reduced by 31% when taking into account the percentage of their time spent on providing SRMNH services. With the exception of midwives and specialist physicians (mainly obstetrician/gynaecologists) most cadres are estimated to spend less than half of their time on SRMNH.

**Fig. 2:**
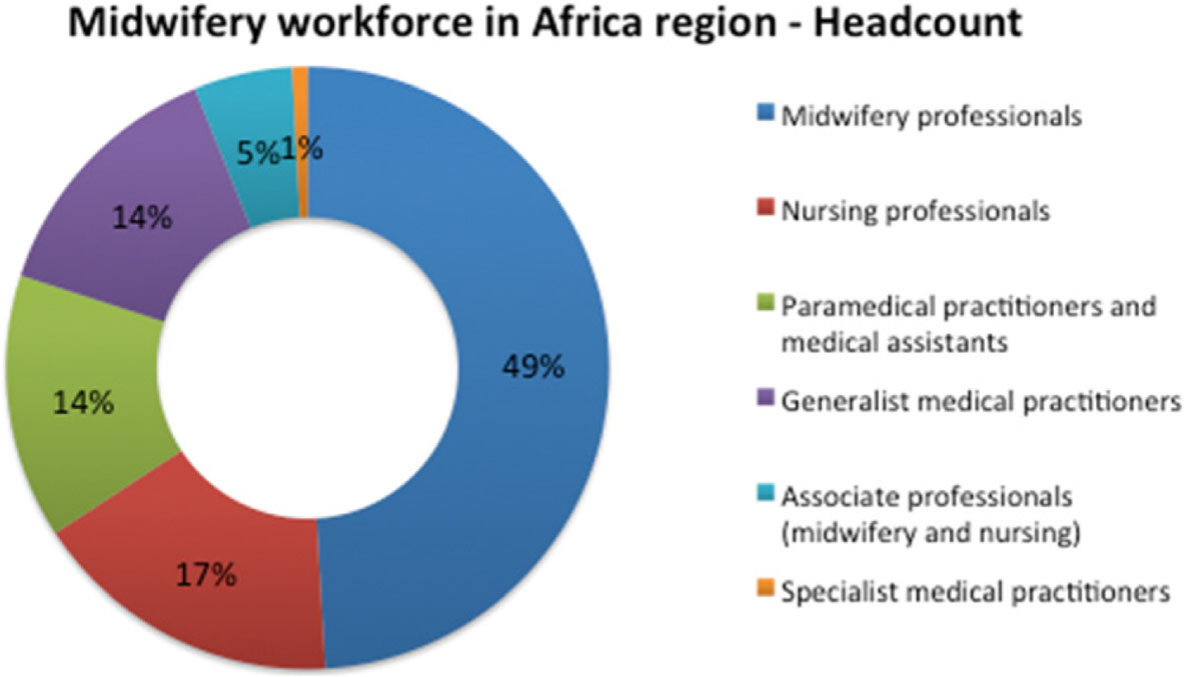
SRMNH workforce in 41 African countries by headcount

**Fig. 3:**
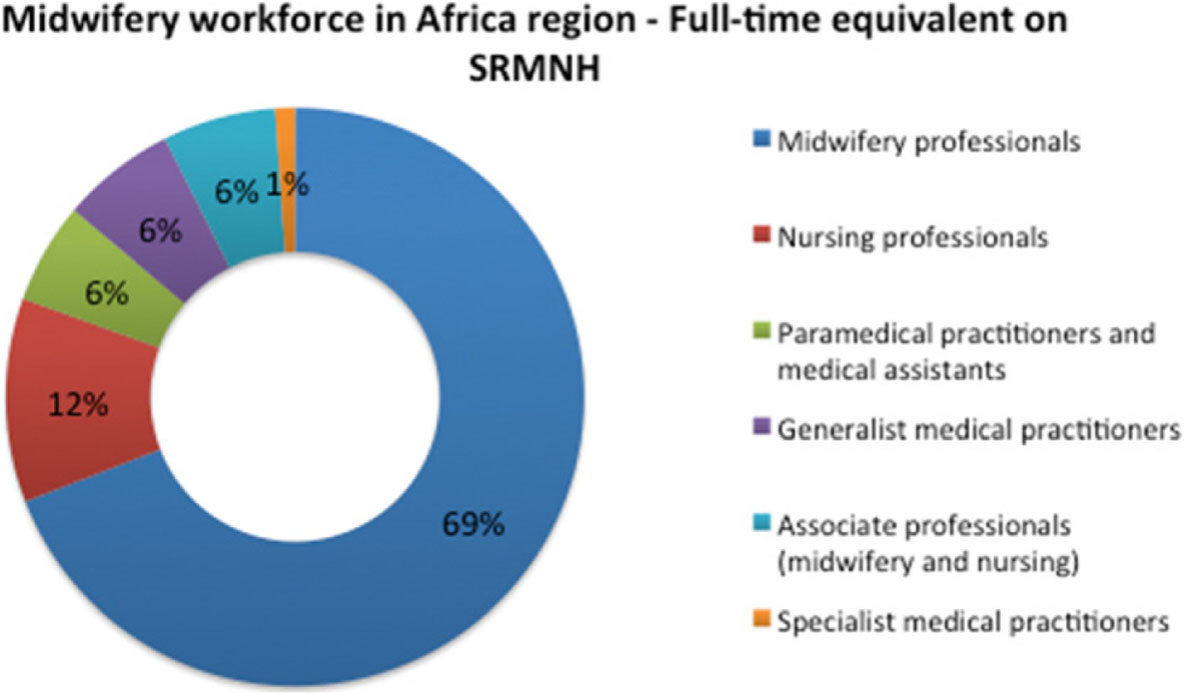
SRMNH workforce in 41 African countries by FTE on SRMNH

Figures 2 and 3 show the distribution of the SRMNH workforce by category. Figure 2 shows the composition of the total workforce by headcount, and Fig. 3 shows the composition by FTE. Headcount alone gives an inflated idea of the contribution by a cadre to SRMNH care if that cadre spends only a small proportion of its working time on SRMNH. Midwifery professionals make up 49% of the SRMNH workforce by headcount and 69% of the workforce when taking into account the percentage time spent on SRMNH, with nursing professionals the second most numerous category, comprising 17% of the workforce by headcount and 12% by FTE on SRMNH.

#### Estimates of potential met need in 2012

In 2012, the 41 countries of the region had an average potential met need of 45%, expected to slightly increase to 51% in 2030.

Not all countries followed the same trend or trajectory for potential met need. Three types of trajectories were identified, based on current (2012) and future (2030) potential met need estimates, and the trajectory direction (whether potential met need was projected to increase or decrease between 2012 and 2030, assuming no change in the current policy situation (Table 1)).

**Table 1:** Country groups by potential met need trajectory from 2012 to 2030

	Group name	Number of countries	Potential met need 2012	Potential met need 2030	Direction
1	Making or maintaining progress	14	Any level	>50% and higher than or equal to potential met need 2012	Increase/No change
2	At risk	6	>50%	Lower than potential met need 2012	Decline
3	Low performing	21	<50%	<50%	Any

The group of countries where potential met need was projected to increase or remain stable between 2012 and 2030, and to be above 50% in 2030, were labelled the “Making or maintaining progress” group. The group of countries where potential met need was above 50% in 2012, and was projected to decline between 2012 and 2030 was labelled “At risk”. Finally, the group where potential met need was below 50% in both 2012 and 2030 was labelled “Low performing”.

According to WHO estimates for 2015 [9], the mean MMR is 504 for countries in the “Making or maintaining progress” group, 370 for countries in the “At risk” group, and 554 for countries in the “Low performing” group. This corresponds with our categorization of the “At risk” group having relatively high potential met need at present, the “Low-performing” group having low potential met need at present, and the “Making or maintaining progress” group presenting a mix between high and low potential met need.

##### “Making or maintaining progress” group

 This group included countries that started at different levels of potential met need in 2012, but potential met need was projected to increase or remain stable so that by 2030 it would be greater than 50%. These countries were: Angola, Burundi, Côte d’Ivoire, Ghana, Kenya, Liberia, Malawi, Niger, Nigeria, Senegal, South Africa, Togo, Tanzania and Zimbabwe.

This group can be separated into two sub-categories, those with potential met need below 50% in 2012, and those with potential met need above 50% in 2012. For those below 50% in 2012, the projected increases in potential met need tended to be large, e.g. Malawi, where potential met need increases from 20% in 2012 to 89% in 2030. The sub-group that started with potential met need above 50% in 2012 comprises Burundi, Liberia, Niger, Nigeria, South Africa, Tanzania and Zimbabwe. According to our modelled projections, the potential met need in these countries was projected to remain as high or increase by 2030.

##### “At risk” group

 The countries in this group were: Comoros, Democratic Republic of Congo (DRC), Gabon, Rwanda, Swaziland and Zambia. They were characterised by a level of potential met need above 50% in 2012, which was expected to decline by 2030.

Four of these countries were expected to face large declines, and therefore considered at “high risk”: DRC (potential met need projected to decline from 53% to 14%), Gabon (from 99% to 67%), Rwanda (from 59% to 42%) and Zambia (from 98% to 48%). For Comoros and Swaziland the declines were much smaller and potential met need remained above 50%, so in the discussion we focus on the “high risk” group.

##### “Low-performing” group

 Half of the countries (21) fell into this category, which was defined by estimated potential met need below 50% in both 2012 and 2030: Benin, Botswana, Burkina Faso, Cameroon, Central African Republic, Chad, Congo, Eritrea, Ethiopia, Gambia, Guinea, Guinea-Bissau, Lesotho, Madagascar, Mali, Mauritania, Mozambique, Sao Tome and Principe, Sierra Leone, South Sudan, Uganda.

Within this group we also observed a sub-group which will potentially report alarming metrics in the region: not only was potential met need very low, but it was projected to decline between 2012 and 2030. These countries were: Burkina Faso, Central African Republic, Congo, Lesotho, Madagascar, Mali, and São Tomé and Principe. The average (mean) projected potential met need in this group in 2030 was just 20%.

#### Alternative policy scenarios

**Fig. 4:**
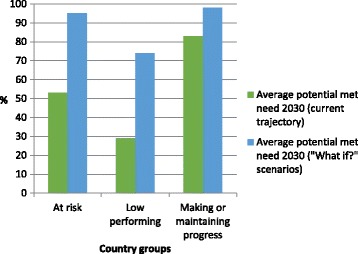
Potential met need in each country group under the current trajectory and alternative policy scenarios (“What if?” scenarios)

By applying the alternative “what if?” policy scenarios (reducing pregnancies by 20% by 2030; doubling the number of midwife, nurse and physician graduates by 2020; improving efficiency by 2% per year until 2030; and halving the rate of voluntary attrition between 2012 and 2017), potential met need in 2030 can rise from an average of 53% to 95% in the “at risk” group, from 29% to 74% in the “low performing” group, and from 83% to 98% in the “making or maintaining progress” group (Fig. 4). However, these will only be possible with very strong political will and substantial investment.

## Discussion

This discussion uses the information from the SoWMy 2014 survey to explore why African countries found themselves on different trajectories with regards to the projected evolution of their workforce and their potential met need estimates, and what policy measures could be put in place to increase potential met need. The significance of potential met need within the wider framework of effective coverage and global human rights principles of universal health coverage and equity is also considered.

Overall, the available SRMNH workforce in the region was not meeting the needs of the population. The region had a low average potential met need in both 2012 and 2030 (average potential met need in 2012 for African region = 45%, other regions = 59%; average potential met need in 2030 for Africa region = 51%, other regions = 72%). This low potential met need was due to an overall deficit in workforce numbers. On the supply side, inflows to the workforce were not large enough to replace the outflows. On the need side, demographic trends also contributed to the lack of projected progress. The average (mean) total fertility rate of the 41 countries is 4.9, compared to 3.0 for the remaining SoWMy countries [31]. Therefore, SRMNH needs in terms of number of women of reproductive age, estimated pregnancies and births are expected to increase.

Socio-economic contexts with increasing inequities and other dimensions of effective coverage such as the quality of education and competencies of SRMNH professionals, not included in the model, also need to be taken into account. With this in mind, the characteristics of the different country groups are discussed below. The discussion also highlights specific barriers and policy solutions relevant to each group. Although certain policies may be applicable to countries in similar situations, they should always be aligned to the specific context and needs of a country or region.

### “Making or maintaining progress” group

As noted above, this group was split into two sub-groups: those with potential met need below 50% in 2012, and those with potential met need above 50% in 2012.

For the group that started below 50%, the projected increases in potential met need were primarily driven by projected increases in the size of the workforce outstripping population growth. While fertility in this sub-group was high, at the same time, all of the countries in this sub-group were projected to see their FTE-adjusted SRMNH workforce increase to at least double its 2012 level by 2030. Crucially, these gains will only be obtained if countries follow through on the commitment to train additional health workers. This may not be guaranteed, as countries in the region frequently lack the necessary resources and support to implement such targets [32]. Beyond achieving high levels of availability, these countries should also focus on ensuring that there is effective coverage of SRMNH services.

Despite having potential met need above 50% in 2012, countries in the other sub-group had MMRs above 300 in 2015 (except South Africa). This may indicate that these countries faced strong challenges in the domains of accessibility, acceptability and quality of SRMNH services, which were not taken into consideration in the potential met need modelling. Further investigation is required to understand exactly why the high numbers of health workers were not delivering effective SRMNH outcomes to the population, and appropriate policies should be formulated in response.

The expansion of availability should never be at the cost of any of these other dimensions, in particular, quality of care. This is increasingly being recognised by policy-makers across the world, many of whom are prioritising respectful and woman-centred care [33].

### At risk group

The fact that these countries managed to achieve relatively high current levels of potential met need indicates that lack of financial resources may have been less of a barrier than in some of the other countries (Gabon and Zambia were upper-middle and lower-middle income respectively, and DRC and Rwanda were low income) [34]– but they must be careful not to follow a policy direction which could undermine their potential to meet the health needs of the population.

The reasons for the projected decline in potential met need can be traced to factors on the demand and supply sides. On the supply side, these countries were projected to see large outflows from the workforce between 2012 and 2030, due to voluntary attrition and retirement (the two main sources of outflows in the model). The age distribution of the workforce can also be a factor: for example, in Rwanda, 60% of GPs were over the age of 40. Based on the policies and programmes in place at the time, these losses were not being replaced with sufficient new graduates, which meant that availability of SRMNH workers was projected to decline in DRC, Gabon and Zambia; and to increase only slightly in Rwanda. Meanwhile, on the demand side, all countries in this group exhibited a very high fertility rate. Rising populations will result in more women of reproductive age, pregnancies and births in these countries, which will raise the demand for SRMNH services.

Policies to increase potential met need can focus on increasing availability and/or reducing need. Countries in the “At risk” group should pay close attention to the future supply into the health workforce: why are the numbers of graduates insufficient? Are students dropping out of education? For some countries, high attrition rates before completing the education programme were reported in the SoWMy 2014 survey: in DRC midwifery students were reported to have a 21% drop-out rate before graduation, and in Gabon, midwives and generalist physicians were reported to have pre-graduation drop-out rates of 40% and 44% respectively. Policies need to focus on enrolling sufficient numbers of students, ensuring that students remain in education, deploying new graduates in a timely manner, and providing incentives and enabling working environments to promote the retention of health workers in the workforce [35–37]. The WHO Roadmap for scaling up HRH in the African region is a useful strategy document [38] that speaks to these requirements. Local level planning, for example through recruitment of students from hard-to-reach or rural areas selected by the community itself based on their needs [39, 40] will minimize attrition and increase deployment and retention rates [41]. Decentralized schools offering culturally sensitive programmes will be able to increase the acceptability of care provided for the respective communities [42]. Decentralized recruitment particularly in poor rural communities [43, 44] and incentive packages but also well-equipped and secure health facilities could increase retention rates of the workforce [45]. Strategic deployment of midwives and other health professionals to ensure a functioning network of EmONC (Emergency Obstetric and Newborn Care) facilities also requires effective local planning. On the need side, policies focused on expanding access to family planning could help to increase met need [46, 47].

### Low-performing group

Countries in the low-performing group were characterised by low numbers of SRMNH workers relative to the size of the population [48]. Apart from Botswana, which was an upper-middle income country, all were low or lower-middle income countries [34].

Most of the low-performing countries were projected to increase the size of the SRMNH workforce, as the number of projected new graduates was expected to be larger than the number leaving the workforce. However, many started from very low numbers of SRMNH workers so the expected increases were not sufficient to meet more than 50% of the need. According to UN Population Division 2015 estimates [49], all of the low performing countries apart from Botswana and Madagascar were expected to increase their number of pregnancies by 2030.

After the data were collected for this study, three ‘low performing’ countries (Guinea, Liberia and Sierra Leone) were severely affected by the Ebola virus disease outbreak. They require additional efforts and resources, as the already limited workforce was severely overstretched in dealing with the emergency, and numerous health workers were affected by the disease. There is evidence that the loss of life among health workers resulted in a reversal of recent progress in maternal and infant mortality, meaning that significant numbers of health workers needed to be recruited immediately [50].

Low-performing countries had low numbers of FTE workers educated in all of the midwifery competencies specified by the ICM [51]. In fact, in 10 of these countries, over half of the FTE-adjusted SRMNH workforce was made up of auxiliary cadres. As these auxiliary cadres did not have the full set of midwifery competencies, these countries suffered in their potential met need estimates.

Despite low levels of potential met need, several of these countries have made significant strides in improving maternal health from a much worse situation in the past. This progress has been achieved through targeted policies and plans in each country, such as the Health Extension Program in 2003 which expanded coverage of Health Extension Workers in Ethiopia [52], the Primary Health Care Policy in Eritrea which promoted health at the local level [53], or the HRH development plans in Mozambique [54]. Substantial long-term investments in infrastructure, education and improvement of working conditions of the health workforce will improve access, especially for vulnerable populations, which suggests that the main focus for international support should be on investing sustainably in initiatives such as these. Such investment involves health system strengthening as part of efforts towards universal health coverage (UHC) [55]. In this regard, including equity measures such as tools and indicators will increase UHC for disadvantaged groups [56]. However, investment in education alone will not be sufficient and will have to be combined with investment in regulation, effective human resource management, and the service delivery environment (EmONC network of facilities and referral mechanisms) in which future midwives will work [57].

The results of the alternative policy trajectory indicate that if certain policy changes can be put into place, many of these countries can achieve remarkable progress by 2030. If countries make efforts to reduce pregnancies, increase graduates, improve efficiency and reduce attrition, potential met need can increase from an average of 29% in 2030 (current trajectory) to an average of 74%, and 11 out of the 21 low-performing countries can achieve a potential met need of 85% or higher in 2030. A detailed analysis of the potential impact of these increases in coverage, in terms of lives saved of women and infants, is beyond the scope of this study. However, there is evidence that even modest increases in coverage of SRMNH services can have a large impact, for countries at all levels of development [58].

Another policy option which was not considered in the original SoWMy study but which could yield great benefits for the countries in this group, given the high percentage of auxiliary workers comprising their workforce, is task-shifting or up-skilling of the auxiliary nursing and midwifery cadres. These policies are part of WHO guidance, and have been successful in increasing coverage of maternal and newborn health services [27].

### Limitations

Potential met need estimates rely strongly on the quality of data on the SRMNH workforce, however reliable, up-to-date workforce data are hard to obtain in income settings where human resources information systems (HRIS) for health are lacking or inadequate [59, 60]. In particular, data on the percentage time that health workers spend on SRMNH services are not frequently collected, which means that assumptions had to be made about this key item of data.

Other assumptions were also used due to missing data. Key amongst these is the principle of economic efficiency which was used to allocate the working time needed by the population to the time available from SRMNH workers. This is not the only possible method of allocation.

Potential met need was estimated as a national average, and did not account for sub-national variations. Countries must consider whether coverage is equitable with a particular focus on reaching vulnerable, marginalized and displaced populations.

Many countries in the region have recently taken part in an exercise to update their SoWMy and a regional midwifery workforce report for East and Southern Africa is forthcoming. It is possible that, since the SoWMy data collection took place, countries in the region have taken additional action affecting the availability of SRMNH workers, in which case this new report will show some different patterns.

## Conclusions

This study has demonstrated that it is possible to create an overall snapshot of the availability of the SRMNH workforce in the African region, and to model how availability may evolve. This methodology yields useful insights into the different situations faced by countries in the region, and points to regional inequities and the need for context-adapted policies. Notwithstanding the potential of this methodology, the need for better workforce data emerges as a fundamental limitation. The analysis depends on 10 essential data items relating to size and composition of the SRMNH workforce [1], yet many countries lacked even these basic data, which made it necessary to use assumptions in their place for the purposes of modelling. The capacity to systematically collect these data at the national level needs to be strengthened. Essential to this process are adequately functioning and resourced HRH observatories [61], and national health workforce accounts, which allow for the standardization of data collection and improve the consistency and regularity of data collected [62]. In a welcome development, the WHO Global Strategy on Human Resources for Health 2030 [63] places these issues into the spotlight, with specific targets for countries. An additional limitation of this analysis is that it focuses on availability, which is just the first dimension of effective coverage of the SRMNH workforce. Strengthening the availability, accessibility, acceptability and quality of the SRMNH workforce is fundamental to meeting the objectives of Sustainable Development Goal 3, and more broadly to guarantee the health and economic development of the population. We hope this paper helps to show how improved workforce data can greatly assist in planning and programming for scaling-up SRMNH services to achieve equity for all.

## Additional file

Additional file 1: Table of key data. (XLSX 12 kb)

## Abbreviations

AAAQ: Availability, accessibility, acceptability and quality
CARMMA: Campaign for Accelerated Reduction of Maternal Mortality in Africa
DRC: Democratic Republic of the Congo
EmONC: Emergency Obstetric and Newborn Care
FTE: Full-time equivalent
HRH: Human resources for health
HRIS: Human resources information systems
ICM: International Confederation of Midwives
MMR: Maternal mortality ratio
SoWMy: State of the World’s Midwifery
SRMNH: Sexual, reproductive, maternal and newborn health
UHC: Universal health coverage
UNFPA: United Nations Population Fund
UNICEF: United Nations Children’s Fund
WHO: World Health Organization
